# Development of a Social Communication Intervention Mobile App for Adolescents With Autism Spectrum Disorder and Social Communication Disorder: Protocol for a Pilot Randomized Clinical Trial

**DOI:** 10.2196/66419

**Published:** 2025-09-09

**Authors:** Enn Young Lee, SungJa Cho, Ran Ju, Hyunjae Kim, Tae Young Choi, Jae Hyun Yoo, Yoo-Sook Joung

**Affiliations:** 1 Neudive Inc Seoul Republic of Korea; 2 Graduate School The Catholic University of Korea Gyeonggi-do Republic of Korea; 3 Department of Psychiatry School of Medicine Daegu Catholic University Daegu Republic of Korea; 4 Department of Psychiatry, Seoul St. Mary's Hospital College of Medicine, The Catholic University of Korea Seoul Republic of Korea; 5 Department of Psychiatry, Samsung Medical Center School of Medicine Sungkyunkwan University Seoul Republic of Korea

**Keywords:** autism spectrum disorder, social communication disorder, digital therapeutics, adolescents, clinical trial, social communication

## Abstract

**Background:**

Autism spectrum disorder (ASD) and social communication disorder (SCD) are neurodevelopmental disorders characterized by deficits in social communication that hinder social adaptation, with limited pharmacological options for therapy owing to the absence of identified biomarkers. Individuals with ASD or SCD require lifelong interventions tailored to their development stages. However, most existing interventions primarily focus on early childhood, leaving adolescents relatively underserved. Moreover, timely access to interventions is often limited by geographic and economic barriers as specialized clinics and therapists tend to be concentrated in major urban areas.

**Objective:**

This pilot randomized clinical trial aimed to evaluate the initial safety and efficacy of NDTx-01, a digital therapeutic (DTx) for adolescents with ASD or SCD. NDTx-01 was designed to overcome the accessibility limitations by integrating cognitive behavioral therapy principles, story-based interventions, and gamification elements.

**Methods:**

We introduce a protocol for a multicenter, prospective, assessor-blinded pilot randomized clinical trial involving children and adolescents aged 10 to 18 years diagnosed with ASD or SCD. Participant enrollment was conducted at 3 major medical hospitals. Enrolled participants were randomly assigned to either the intervention group (NDTx-01 and treatment as usual [TAU]) or the control group (TAU only). TAU included medications and therapeutic services. Participants were instructed to use the app approximately 10 minutes per day, 5 days a week. To evaluate the efficacy of NDTx-01, standardized tools were administered, including the Korean version of the Vineland Adaptive Behavior Scales, Second Edition (K-VABS-II); the Social Responsiveness Scale, Second Edition; Clinical Global Impressions–Severity (CGI-S); Clinical Global Impressions–Improvement (CGI-I); the Social Communication Questionnaire; and the Korean version of the Stress Index for Parents of Adolescents. Assessments were conducted in weeks 0, 1, 2, 4, and 6, except for K-VABS-II, CGI-S, and CGI-I, which were administered only in weeks 0, 4, and 6. Statistical analyses were conducted using the SAS software. Between-group differences were assessed using independent 2-tailed 2-sample *t* tests or Wilcoxon rank sum tests. Within-group changes were evaluated using paired *t* tests or Wilcoxon signed rank tests.

**Results:**

From August 2024 to December 2024, a total of 42 individuals were screened, 39 (93%) participants who met the inclusion criteria were enrolled, and 1 participant withdrew consent; the remaining participants completed the study. The pilot randomized clinical trial was successfully completed, and the results were published in April 2025. As of 2025, we are conducting a confirmatory clinical trial at 5 major hospitals across South Korea.

**Conclusions:**

The results of this pilot clinical trial provided important insights into the initial safety and efficacy of DTx as interventions for adolescents with ASD and SCD. Both groups demonstrated statistically significant improvements in adaptive skills and socialization.

**Trial Registration:**

Clinical Research Information Service KCT0009140; https://cris.nih.go.kr/cris/search/detailSearch.do?seq=26713

**International Registered Report Identifier (IRRID):**

DERR1-10.2196/66419

## Introduction

### Background

Autism spectrum disorder (ASD) is a neurodevelopmental disorder characterized by deficits in social communication, repetitive behavior, and restricted interests [1]. Deficits in social communication result in difficulties in engaging in reciprocal interactions. These challenges manifest in various situations, such as maintaining dialogue, comprehending humor and idioms, and understanding others’ emotions. These impediments play a critical role in the complexity of social adaptation.

Social communication disorder (SCD) is a new diagnosis included in the fifth edition of the *Diagnostic and Statistical Manual of Mental Disorders* [1]. While individuals with SCD share the primary impairment of deficits in social communication with those with ASD, they can be differentiated from those with ASD in that people with SCD do not manifest restricted interests and repetitive behaviors [1-4]. As science develops and social awareness increases, there has been an observed increase in the diagnostic rates and prevalence of both SCD and ASD. The Centers for Disease Control and Prevention [5] reported that 1 in 44 people have ASD in the United States. According to Kim et al [6,7], the prevalence of ASD in South Korea is estimated to be 2.64% (95% CI 1.91%-3.37%), which translates to 1 out of 38 people, and Russell et al [8] state that the diagnosis rate of ASD in the United Kingdom increased by 787% in the last 2 decades. The prevalence of SCD is difficult to determine because of inconsistent or ambiguous definitions [9]. However, Ellis Weismer et al [4] reported that the prevalence of SCD in developmental disorders was 7% to 11% from 1491 samples in phases 1 and 2 of the Study to Explore Early Development.

To date, there is no specific medication licensed for the treatment of the core symptoms of ASD and SCD as biomarkers have not been fully identified. Face-to-face interventions are recommended by various clinical guidelines, such as *Post-diagnostic Management and Follow-up Care for Autism Spectrum Disorder* [10] and specific interventions for the core features of ASD [11] and SCD [9].

Individuals with ASD and SCD require lifelong care involving various treatments, such as social skill training, speech therapy, applied behavior analysis, and special education. Research shows that approximately 50% of children with ASD have used at least one medication in the past year [12-16], and they are currently undergoing an average of 6 to 8 treatments [17]. Given the critical importance of early intensive intervention for later developmental outcomes [18,19], early diagnosis and adequate treatment are crucial. However, the concentration of medical institutions and treatment services in urban areas poses a significant barrier for individuals living in rural areas seeking proper care [20-22].

The high costs associated with treatment can also pose a burden on individuals, families, and society, potentially impeding continuous interventions [6,7,23]. In the United States, Leigh and Du [24] estimated the total national economic burden of ASD to be US $268 billion in 2015, a figure projected to increase to US $461 billion by 2025. This estimate includes direct medical costs, direct nonmedical costs, and productivity losses. MacKay et al [25] reported an annual cost of £2.3 billion (US $3.1 million) in Scotland. However, it is crucial to recognize that these figures may not fully capture the entire economic burden both within and outside medical institutions and that many treatments fall outside the scope of health insurance coverage.

In several studies, technology-based interventions have proven effective in teaching and developing social skills in patients with ASD and other developmental disorders. Gamification—applying game elements and game design techniques in nongaming contexts [26]—enhances the sense of engagement, immediate feedback, and success in overcoming challenges [27]. This can significantly increase the effectiveness of technology-based interventions. Repetitive learning and training in a safe environment, such as using smart devices at home, can reduce anxiety and distraction in adolescents in need [28-30]. Furthermore, technology-based interventions can lessen the physical, temporal, and economic burdens on caregivers.

The prevalence of chronic diseases and the high cost of face-to-face medical treatments underscore the need for digital therapeutics. According to Statista [31], the digital therapeutics market will increase from US $1.67 billion in 2016 to US $8.94 billion in 2025. The number of DTx users is projected to grow to 652.4 million by 2025 from 44 million in 2021. The global market value of digital therapeutics was US $4.2 billion in 2021 and is estimated to reach US $17.3 billion by 2030 following the impact of the COVID-19 pandemic.

### Objectives

The development of digital therapeutics for social skill training holds significant potential but is still in its early stages. Consequently, there is limited guidance on how to evaluate the efficacy and safety of newly developed digital social skill training programs, especially for ASD and SCD. Therefore, we developed a digital therapeutic agent, NDTx-01, and designed a pilot clinical trial to validate its initial safety and efficacy. This pilot clinical trial was conducted in accordance with protocol ND-01 (version 7.1; December 6, 2023). Thus, the research objectives of this study were to design and develop a simulation game–based digital therapy aimed at enhancing social communication and interaction skills for adolescents with ASD and SCD and to develop a pilot clinical trial protocol for assessing the clinical effectiveness of the simulation game–based digital therapy.

## Methods

### Study Design

To improve the social abilities of adolescents with ASD and SCD, we developed a digital therapeutic named NDTx-01. To evaluate the initial efficacy and safety of NDTx-01, a multicenter, prospective, randomized, and assessor-blinded pilot study was conducted.

Participants were instructed to maintain their usual care, such as medication, other face-to-face interventions, and special education, from 4 weeks before the start of the clinical trial to its conclusion. Any changes in their usual care led to participant exclusion from the trial. However, all data collected up to the point of exclusion were preserved in the electronic data capture system for analysis.

**Figure 1:**
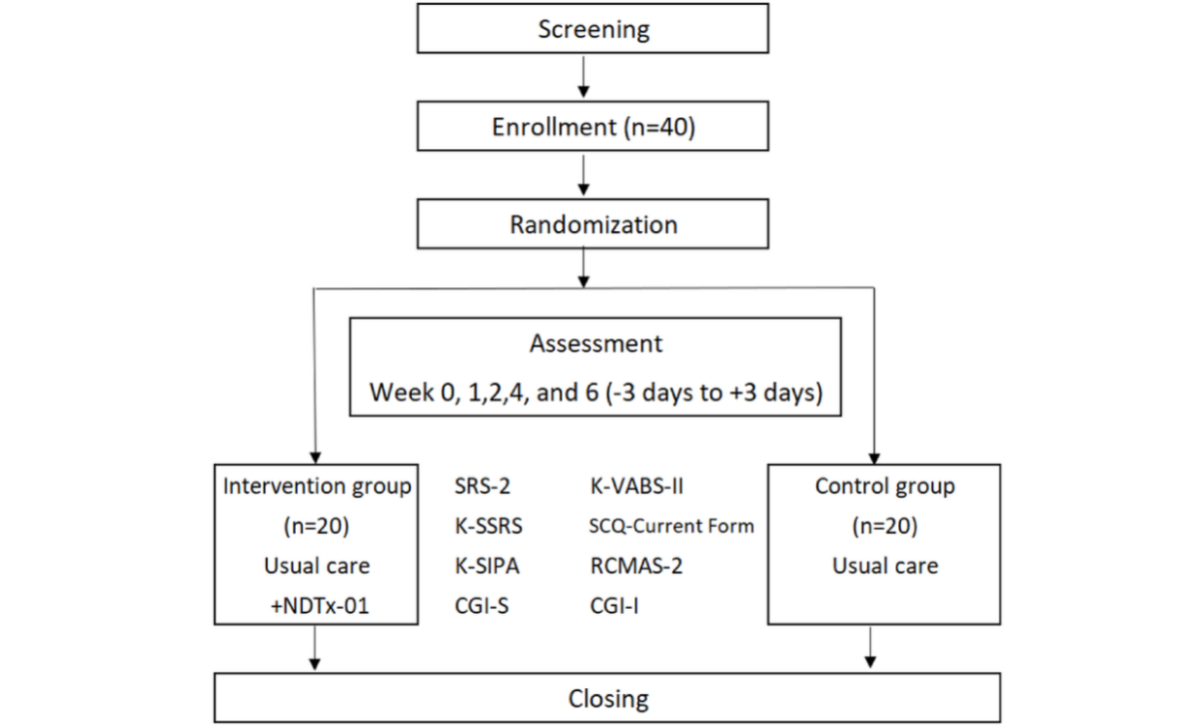
Flowchart of the pilot clinical trial. CGI-I: Clinical Global Impressions–Improvement; CGI-S: Clinical Global Impressions–Severity; K-SIPA: Korean version of the Stress Index for Parents of Adolescents; K-SSRS: Korean version of the Social Skills Rating System; K-VABS-II: Korean version of the Vineland Adaptive Behavior Scales, Second Edition; RCMAS-2: Revised Children’s Manifest Anxiety Scale, Second Edition; SCQ–Current Form: Social Communication Questionnaire–Current Form; SRS-2: Social Responsiveness Scale, Second Edition.

The flowchart of the pilot clinical trial is presented in Figure 1. The intervention group was provided with NDTx-01, which contained stories with questions about social situations commonly experienced in school settings. Questions regarding verbal or nonverbal expressions, appropriate communication responses, and social cognition of the situations they observed were presented, all guided by instructions. At the end of each story, summaries, additional questions, and behavioral tasks were presented. Each session lasted approximately 10 minutes, and the participants used NDTx-01 a total of 5 days a week for 6 weeks. In addition, usual care was maintained for the intervention group participants throughout these 6 weeks. The control group maintained their usual care, or treatment as usual, for 6 weeks, with the option to use NDTx-01 after the end of the clinical trial. Treatment as usual included ongoing care such as pharmacological treatment, speech therapy, psychiatric consultations, or school-based counseling. These interventions needed to be initiated at least 4 weeks before screening and were required to be maintained throughout the study period. The type and frequency of interventions varied based on individual clinical needs and were not standardized by the study protocol.

Assessments were conducted in weeks 0, 1, 2, 4, and 6 or weeks 0, 4, 6 (with a margin of −3 days to +3 days for each) to evaluate the effectiveness of NDTx-01.

### Ethical Considerations

This study underwent thorough ethical review and was approved by the institutional review boards of several medical institutions: Samsung Medical Center (SMC2023-05-070-011), the Catholic University of Korea Seoul St. Mary’s Hospital (KC23DIDS0453), Daegu Catholic University Medical Center (MDCR-23-008-L), and Dankook University Hospital (DKUH 2022-11-012-002). All procedures were conducted in accordance with the relevant guidelines and regulations.

Written informed consent was obtained from all participants by investigators before enrollment. For children and adolescents aged 10 to 12 years, age-appropriate explanations regarding the clinical trial were provided, after which they signed a child-specific informed consent form. Their legal guardian also received detailed information about the clinical trial and signed the standard informed consent form to authorize their child’s participation. Participants aged 13 to 18 years provided assent and signed the informed consent form alongside their guardians in accordance with institutional review board guidelines. Any secondary analyses using existing data for future research purposes will not require additional consent.

All data collected during the clinical trial were securely stored as paper documents and electronic records. To ensure the privacy and confidentiality of participants, data were anonymized and deidentified to prevent personal identification. Data were used solely for clinical trial purposes. Access to source data was restricted to regulatory authorities or for the purpose of the device approval process for NDTx-01; even in such cases, all personally identifiable information remained protected through deidentification protocols. No images were collected, and individual participants cannot be identified from the data obtained.

To compensate for the time and effort required for participation, each participant received KRW 50,000 (approximately US $35) per in-person visit. Compensation was not provided for televisits.

### Study Participants

Participant recruitment took place at the Samsung Medical Center, the Catholic University of Korea Seoul St. Mary’s Hospital, and Daegu Catholic University Medical Center. A total of 40 participants were expected to be enrolled in the study.

As this clinical trial was a pilot study, a formal sample size calculation was not mandatory. However, to inform the design of a confirmatory randomized clinical trial, we aimed to provide preliminary evidence regarding sample estimation. A power of 90% was set for reference purposes [32], and relevant previous studies were reviewed to ensure that the protocol was as evidence based as possible [33-36]. On the basis of these considerations, a total sample size of 40 participants was determined to be appropriate for this pilot randomized clinical trial.

Inclusion criteria were as follows: children and adolescents in the age range of 10 to 18 years diagnosed with ASD or SCD by psychiatrists based on the *Diagnostic and Statistical Manual of Mental Disorders* criteria and assessed using the Autism Diagnostic Interview–Revised (ADI-R) [37], Korean version of the Wechsler Intelligence Scale for Children–Fourth Edition (K-WISC-IV) or Fifth Edition (K-WISC-V) [38,39], and clinical evaluation; adolescents who could use NDTx-01 independently or with caregiver assistance; adolescents who could continue using NDTx-01 for the recommended period; adolescents who agreed not to initiate new additional interventions, including applied behavior analysis or social, educational, or rehabilitation programs from 4 weeks before the start of the clinical trial to its completion; adolescents and their parents who voluntarily agreed to participate in the pilot clinical trial; and adolescents and their parents who could adhere to the clinical protocol.

Exclusion criteria were as follows: adolescents with clinically significant behavioral problems, emotion control problems, psychotic symptoms, or probability of harm (including self-harm); adolescents with serious acute or chronic medical or psychiatric diseases; adolescents who had had serious physical injury or surgery within the previous month; adolescents with serious neurological disorders (ie, brain lesions and mental illnesses); adolescents who were participating in other clinical trials or had done so within the previous 30 days; adolescents who had changes in use or dosage of medications or alterations in participation in therapeutic, educational, or rehabilitation programs that could significantly influence social abilities within 4 weeks before the clinical trial; and adolescents whom the examiner judged inappropriate for the clinical trial owing to ethical concerns or the potential of factors that would influence the trial results. In the event of trial-related harm, participants were entitled to appropriate medical treatment and financial compensation in accordance with the insurance coverage arranged by the sponsor.

### Diagnosis Standardization Education

The ADI-R served as one of the diagnostic assessment tools for participant inclusion. The ADI-R is a semistructured, standardized interview designed to assess ASD symptoms through caregiver reports. While its semistructured format provides flexibility in how questions are explored, it requires administration by a well-trained and experienced assessor. The reliability and consistency of the ADI-R depends not only on its structured framework but also on the assessor’s ability to elicit detailed and developmentally relevant information from caregivers. To maintain the interreliability of the ADI-R assessment, ADI-R training by an internationally certified trainer was conducted, including theoretical lectures, clinical practice, and scoring practice.

To ensure the accuracy and consistency of SCD diagnosis, we convened an SCD diagnosis standardization consensus meeting. The participants included principal investigators and pediatric psychiatrists from all the medical hospitals involved in the pilot clinical trial. During the meeting, specialists shared their clinical diagnostic interpretations.

### Randomization and Assessor-Blinded Approach

For random assignment in this clinical trial, an interactive web response system was used to allocate participants to either the intervention or control group. A stratified block randomization method was applied at each medical center with an allocation ratio of 1:1. A qualified statistician determined the block size, set at 4, and selected seed numbers for the randomization process. This method was chosen to maintain the integrity of the randomization and minimize potential biases in participant allocation.

An assessor-blinded approach was used to ensure the objectivity of assessment during the clinical trial. The assessors responsible for conducting the validation assessments were kept unaware of the group allocation of each participant. To maintain the integrity of this blinding, direct interaction between the assessors and participants, as well as their caregivers, was restricted throughout the assessment process. Instead, clinical research coordinators acted as intermediaries.

### Intervention

The digital therapeutic, NDTx-01, as an intervention tool integrated social skill training and cognitive behavioral therapy methodologies and techniques. Its framework encompassed stages from assessment and reconceptualization to skill acquisition, skill consolidation, and application training to generalization, cognitive processing, and modeling. The social situations presented in NDTx-01 were constructed based on well-verified social skill training programs such as the Program for the Education and Enrichment of Relational Skills (PEERS) and Social Stories [3,33,40-45]. However, existing programs often target different age groups, such as early childhood or early adulthood, which limited their applicability to our intended population. This gap led us to conduct a focus group interview (FGI). A total of 10 individuals participated in the FGI, including 4 (40%) individuals with ASD (n=3, 75% male and n=1, 25% female), 2 (20%) mothers of adolescents with ASD, 1 (10%) special education teacher, 1 (10%) middle school teacher, and 2 (20%) therapists.

Through this FGI, we identified common situations that children and adolescents with ASD find most challenging or distressing in daily school lives or peer relationships. It also provided insights into which social skills were perceived as most critical for social functioning and the underlying reasons for these priorities as expressed by parents, teachers, and therapists.

On the basis of both the literature review and FGI findings, we constructed scenarios and items that reflected social skills applicable to the real lives of children and adolescents with ASD or SCD.

NDTx-01 is a story-based intervention centered on a main player character and 6 nonplayer peers. To design visually relatable and appealing characters, a preference survey was conducted among prospective users. The most favored styles and imagery were then handed to a designer to create the characters, backgrounds, and props. The gamified app was developed using Unity (Unity Technologies) by a developer with expertise in mobile and computer game development. At each developmental stage, detailed discussions were held to ensure the appropriateness of the social scenarios, dialogue content, level of difficulty, and user engagement.

A pilot version of the app underwent usability testing, which evaluated the clarity of the storylines, playability, and overall appeal. The test involved individuals with ASD, their parents, and therapists. The feedback collected from these assessments was used to refine and improve NDTx-01.

A key feature of NDTx-01 was its emphasis on generalization, facilitated by the user’s engagement with diverse situations and scenarios assisted and prompted by direct and sometimes indirect therapeutic interventions in school settings. This construction was particularly relevant because most user groups were affiliated with schools.

**Figure 2:**
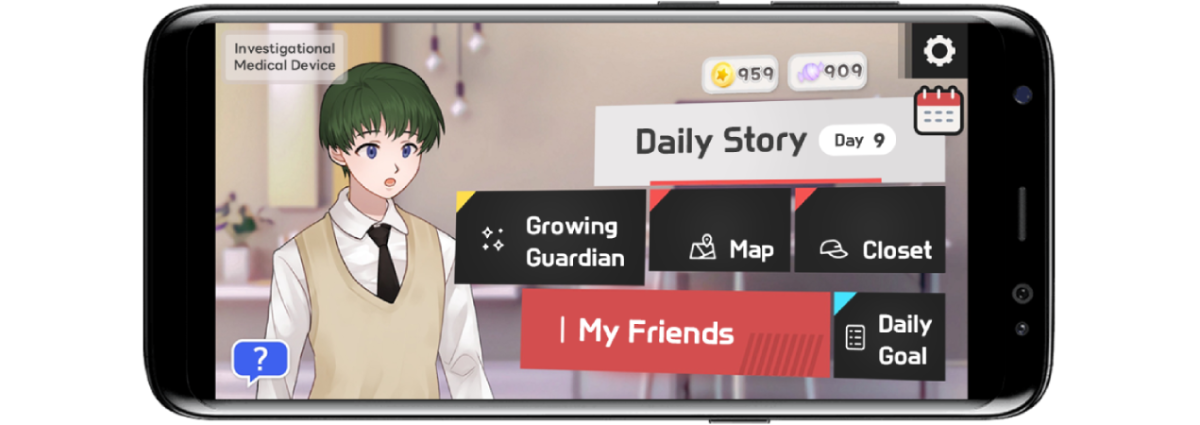
Main menu of NDTx-01. The Daily Story presents 30 stories to last for 6 weeks, 5 days a week.

To aid in the understanding and interpretation of social cues and situations, NDTx-01 provided a broad spectrum of social scenarios and narratives within a school setting, such as making new friends at the start of a new semester and expanding relationships with schoolmates. These scenarios, presented as a Daily Story in the main menu of NDTx-01, run for 10 minutes per day, 5 days a week over 6 weeks (Figure 2). The stories were presented from a third-person perspective to enhance users’ comprehension and perspective-taking abilities. NDTx-01 also featured a supportive fairy and friends who assisted the main character both verbally and nonverbally. This multifaceted intervention not only aided in social skill development but also enhanced user engagement and practical applications in real-world situations. The specific contents and goals of each NDTx-01 session are outlined in Table 1.

**Table 1:** Contents and goals of each session within NDTx-01.

Week and session number			Contents		Goal of the session
**Week 1**					
	1	Day before starting a new grade		Preparing for a new grade emotionally and cognitively	
	2	First day in the classroom		Greetings and a short conversation with a new classmate	
	3	Lunchtime at the school cafeteria		Appropriate topics to talk about with a classmate	
	4	Meeting new classmates in the art room		Expressing interest properly	
	5	One-on-one meeting with the teacher		Appropriate topics to talk about with a teacher	
**Week 2**					
	6	Accidentally hurting a classmate in gym class		How to talk and behave in an embarrassing situation	
	7	Small talk about K-pop stars		Sharing one’s interests with others	
	8	Cleaning time after class		Solving a conflict	
	9	April Fool’s day happening in class		Joining a group activity	
	10	Watching a classical performance		Etiquette to keep at the art theater	
**Week 3**					
	11	Exchanging defective goods at a shop		How to talk and behave when exchanging goods	
	12	Meeting the sister of a friend		How to talk to and behave with an acquaintance of a friend	
	13	Going to the library		Etiquette to keep at the library	
	14	Preparing a birthday present		How to prepare a present for someone	
	15	White lies		Situations that need white lies	
**Week 4**					
	16	Shopping with friends		How to give advice nicely	
	17	A psychological test		Using metaphors to explain a person’s character	
	18	Too much desire to win		Behaviors that can make others uncomfortable	
	19	A presentation		Dealing with social anxiety	
	20	A musical performance		Dealing with performance anxiety	
**Week 5**					
	21	Sharing lunch		Dining etiquette	
	22	Working as a team		Cooperating with others	
	23	Deep down inside one’s mind		Understanding discordance between situations and thoughts	
	24	Language class		Understanding proverbs and idioms	
	25	Unexpected incident		Controlling bad feelings and accepting advice	
**Week 6**					
	26	Helping a friend with shopping		How to express gratitude	
	27	Situations that need to be rejected		How to reject nicely	
	28	Going to the movies		Movie etiquette	
	29	Interviewing others		How to ask questions and respond properly	
	30	Closing		Closing	

### Assessment

#### Assessment Tools for Screening

##### Korean Version of the ADI-R

The ADI-R is a standardized, semistructured tool designed to gather comprehensive information for the assessment of ASD. A skilled clinician conducts in-depth interviews with a guardian familiar with the participant’s developmental history and current daily functioning, such as the mother, grandmother, or other primary caregiver. This approach ensures a detailed understanding of the participant’s developmental milestones and current adaptive functioning, which is critical for accurate ASD assessment. The type of variable is continuous, and a higher score means more symptoms of ASD. The ADI-R was used once to facilitate the selection of participants.

##### K-WISC-IV and K-WISC-V Tools

The K-WISC-IV and K-WISC-V are standardized intelligence tests that measure the intellectual abilities of children and adolescents in the age range of 6 years, 0 months to 16 years, 11 months. The K-WISC-IV analyzes intelligence in full-scale intelligence quantity (FSIQ) and 4 domains, whereas the K-WISC-V presents FSIQ and 5 domains. The type of variable is continuous, and a higher FSIQ score indicates better intellectual ability. The K-WISC-IV or K-WISC-V was used once to facilitate the selection of participants.

#### Assessment Tools for Efficacy and Safety

##### Overview

**Figure 3:**
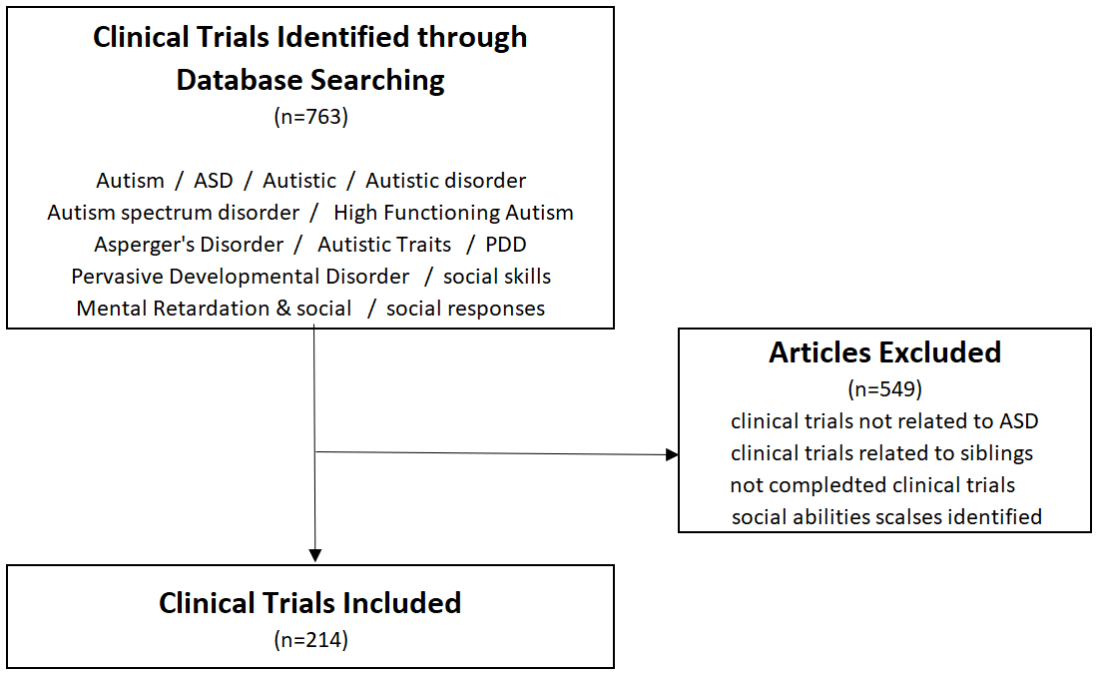
Flow description of the selection process for the literature review. ASD: autism spectrum disorder; PDD: pervasive developmental disorder.

To identify appropriate assessment tools for evaluating the efficacy and safety of treatments for ASD and SCD, we reviewed clinical guidelines and comparable study designs. Scales recommended by the European Medicines Agency, the National Institute for Health and Care Excellence of Great Britain, and the Canadian Paediatric Society were reviewed. An extensive review of clinical trial designs was conducted in November 2022. A search on ClinicalTrials.gov using keywords such as “social skill training,” “intervention,” “ASD,” “Asperger’s syndrome,” “pervasive developmental disorder,” and “mental retardation” yielded 28% (214/763) of trials that met our specified criteria (Figure 3).

As shown in Table 2, among the 214 clinical trials, the Social Responsiveness Scale was used in 108 (50.5%) cases, constituting the highest rate. The Vineland Adaptive Behavior Scales (VABS) was used in 28% (60/214) of cases, and the Social Skills Rating System was used in 5.1% (11/214) of cases. As previously mentioned, the European Medicines Agency recommends the Childhood Autism Rating Scale; Social Responsiveness Scale, Second Edition (SRS-2); VABS, Second Edition; Children’s Yale-Brown Obsessive Compulsive Scale for children with ASD; and Clinical Global Impressions–Improvement (CGI-I). On the basis of the study results, the following assessment tools were selected (Table 3): the SRS-2; the Korean version of the VABS, Second Edition (K-VABS-II); the Korean version of the Social Skills Rating System (K-SSRS); the Social Communication Questionnaire–Current Form (SCQ–Current Form); and the CGI-I. In addition, the Revised Children’s Manifest Anxiety Scale, Second Edition (RCMAS-2), was incorporated to measure anxiety levels among the participants. The Korean version of the Stress Index for Parents of Adolescents (K-SIPA) was used to assess anxiety levels in the parents of the participants. The Clinical Global Impressions–Severity (CGI-S) was also included to assess the clinicians’ brief impressions of the severity of the illness.

**Table 2:** Use rate of the scales among the clinical trials from our literature search (N=214).

Scale	Studies, n (%)
SRS^a^ [46]	108 (50.5)
VABS^b^ [47]	60 (28)
SSRS^c^ [48]	11 (5.1)
SCQ^d^ [49]	8 (3.7)
CCC^e^ [50]	5 (2.3)
ESCS^f^ [51]	5 (2.3)
BOSCC^g^ [52]	4 (1.9)
TSSK^h^ [53]	3 (1.4)
GSOM^i^ [54]	2 (0.9)
SSiS^j^ [55]	2 (0.9)
Others	6 (2.8)

^a^SRS: Social Responsiveness Scale.

^b^VABS: Vineland Adaptive Behavior Scales.

^c^SSRS: Social Skills Rating System.

^d^SCQ: Social Communication Questionnaire.

^e^CCC: Children’s Communication Checklist.

^f^ESCS: Early Social Communication Scales.

^g^BOSCC: Brief Observation of Social Communication Change.

^h^TSSK: Test of Social Skills Knowledge.

^i^GSOM: General Social Outcome Measure.

^j^SSiS: Social Skills Improvement System.

**Table 3:** Assessment schedule.

Outcome measures	Visit 1^a^ (screening)—−4 wk approximately	Visit 2^a^ (baseline)—day 0	Visit 3 (tele-visit 1)—day 7 (–3 d to +3 d)	Visit 4 (tele-visit 2)—day 14 (–3 d to +3 d)	Visit 5—day 28 (–3 d to +3 d)	Visit 6 (final visit)—day 42 (–3 d to +3 d)
K-VABS-II^b^		✓			✓	✓
SRS-2^c^		✓	✓	✓	✓	✓
K-SSRS^d^		✓	✓	✓	✓	✓
RCMAS-2^e^		✓	✓	✓	✓	✓
K-SCQ^f^		✓	✓	✓	✓	✓
K-SIPA^g^		✓	✓	✓	✓	✓
CGI-S^h^		✓			✓	✓
CGI-I^i^					✓	✓

^a^Visits 1 and 2 can be conducted on the same day.

^b^K-VABS-II: Korean version of the Vineland Adaptive Behavior Scales, Second Edition.

^c^SRS-2: Social Responsiveness Scale, Second Edition.

^d^K-SSRS: Korean version of the Social Skills Rating System.

^e^RCMAS-2: Revised Children’s Manifest Anxiety Scale, Second Edition.

^f^K-SCQ: Korean version of the Social Communication Questionnaire.

^g^K-SIPA: Korean version of the Stress Index for Parents of Adolescents.

^h^CGI-S: Clinical Global Impressions–Severity.

^i^CGI-I: Clinical Global Impressions–Improvement.

##### SRS-2 Instrument

The SRS-2 is a quantitative measure of symptoms associated with ASD. It comprises 5 distinct subscales: social awareness, social cognition, social communication, social motivation, and restricted interests and repetitive behavior. The scale consists of 65 items, with each item rated on a 4-point scale to reflect symptom severity. The scores for each subdomain and the total score were aggregated and converted into T scores using a standardized scoring method. Higher scores indicate more serious symptoms of ASD. The SRS-2 was administered in weeks 0, 1, 2, 4, and 6 (−3 days to +3 days for each).

##### K-VABS-II Instrument

The K-VABS-II [56] measures adaptive behavior levels across 4 domains (communication, daily living skills, socialization, and motor skills) and provides an adaptive behavior composite score. Higher standard scores signify more adaptive behavior. The K-VABS-II was administered in weeks 0, 4, and 6 (−3 days to +3 days for each).

##### K-SSRS Secondary Level, Student Form

The K-SSRS [57] is an assessment tool designed to evaluate adolescents’ social skills. It comprises 39 items organized to assess proficiency in 4 subdomains: empathy, assertion, self-control, and cooperation. Higher average scores on the 4 domains and total scale indicate greater proficiency in social skills. The K-SSRS was administered in weeks 0, 1, 2, 4, and 6 (−3 days to +3 days for each).

##### SCQ–Current Form

The Korean version of the SCQ–Current Form [58] is a parent-reported assessment tool designed to screen for ASD and its associated symptoms. This tool comprises 40 items extracted from the ADI-R. Caregivers answer *yes* or *no* to each item based on the observed behaviors of the patient over the previous 3 months. A higher score indicates a higher probability of ASD. The SCQ–Current Form was administered in weeks 0, 1, 2, 4, and 6 (−3 days to +3 days for each).

##### RCMAS-2 Instrument

The Korean version of the RCMAS-2 [59] is a self-report tool designed to evaluate the level and characteristics of anxiety experienced by children and adolescents. It comprises 49 items, each requiring a simple *yes* or *no* response.

The tool includes an inconsistent response index and defensiveness scale, which function as validity scales. When the scores on these validity scales fall within the normal range, the resulting measures of total anxiety, physical anxiety, worry, and social anxiety are deemed credible and reflective of the respondents’ anxiety levels. Higher scores mean higher anxiety levels. The RCMAS-2 was administered in weeks 0, 1, 2, 4, and 6 (−3 days to +3 days for each).

##### K-SIPA Instrument

The K-SIPA [60] is a comprehensive assessment tool designed to measure parental stress associated with raising adolescents. It comprises 112 items that respondents are required to answer on 5-point and 2-point Likert scales. It evaluates parental stress across 5 domains: adolescent domain, parent domain, adolescent-parent relationship domain, life stress domain, and an index of total parenting stress. Both the adolescent and parent domains are divided into 4 subdomains. Higher scores on the K-SIPA indicate greater levels of parental stress. The K-SIPA was administered in weeks 0, 1, 2, 4, and 6 (−3 days to +3 days for each).

##### CGI-S and CGI-I Instruments

The Clinical Global Impressions provides a brief assessment of a clinician’s perspective on a patient’s global functioning. The CGI-S assesses the severity of a patient’s illness, whereas the CGI-I assesses the degree of improvement of symptoms. Higher scores indicate worse conditions. The CGI-S was administered in weeks 0, 4, and 6 (−3 days to +3 days for each). The CGI-I was administered in weeks 4 and 6 (−3 days to +3 days for each).

### Statistical Analysis

The data collected in the pilot clinical trial were analyzed using the SAS statistics program (SAS Institute Inc). Initially, descriptive analysis was applied to the entire dataset. A frequency analysis was conducted on demographic data. Outcome measures were analyzed based on the full analysis set.

For within-group comparisons, the analytical approach was contingent on the distribution of the data. If the data were normally distributed, a paired *t* test was used to compare mean values. For data that were not normally distributed, the Wilcoxon signed rank test was used. If the bilateral significance probability was <.05, within-group changes were considered statistically significant.

For between-group comparisons, when the data were normally distributed, an independent 2-sample *t* test was applied to analyze the differences in means. Otherwise, the Wilcoxon rank sum test was conducted. If the bilateral significance probability was <.05, it was considered statistically significant. Missing data were imputed using the last observation carried forward method.

## Results

This clinical trial was registered with the Clinical Research Information Service, which is part of the World Health Organization International Clinical Trials Registry Platform, under the number KCT0009140 on July 7, 2023. The Korean Food and Drug Administration approved this clinical protocol in February 2023. A total of 42 participants were screened, and 39 were enrolled in the study. As of August 2024, this clinical trial was completed, and the clinical study report was submitted to the Ministry of Food and Drug Safety in April, 2024 The findings and outcomes of this pilot randomized clinical trial were published in April 2025 [61].

## Discussion

### Expected Findings

The NDTx-01 is a newly developed digital therapeutic tool designed to improve social communication and interaction abilities in adolescents with ASD and SCD. On the basis of cognitive behavioral therapy principles, social skill training, story-based interventions, and gamification elements, NDTx-01 aims to deliver comprehensive therapeutic stimuli and interventions to achieve these therapeutic objectives. To evaluate its efficacy, we planned to conduct a pilot randomized clinical trial using a stringent protocol. The goal was to substantiate the effectiveness of NDTx-01 in improving the social skills and adaptability of adolescents with ASD and SCD.

From a therapeutic perspective, NDTx-01 was anticipated to serve as an effective stand-alone intervention for enhancing social communication and cognitive abilities in adolescents with ASD and SCD [61]. In addition, its potential as a supplementary tool to traditional face-to-face interventions was also noteworthy. The diverse situational simulations and story-based formats of NDTx-01 were designed to foster broader generalization of learned skills, offering a multifaceted approach to treatment.

From a socioeconomic perspective, NDTx-01 is expected to serve as a viable alternative to conventional clinical interventions for individuals with ASD and SCD, particularly in terms of accessibility. This digital therapy aims to bridge the gap in access to high-quality medical treatments, potentially democratizing the availability of effective therapies for these populations [62-65].

However, this study has some limitations. Our methodology involved reviewing clinical trials from ClinicalTrials.gov focusing on the assessment tools used for ASD and related developmental disorders in adolescents and targeting their social cognition ability. We selected the K-VABS-II, SRS-2, K-SSRS, RCMAS-2, SCQ–Current Form, K-SIPA, CGI-S, and CGI-I to evaluate the effectiveness of NDTx-01. While these tools are widely accepted, it is important to note that none are officially designated as diagnostic assessments for ASD or SCD. In addition, the SRS-2 and K-SSRS have not been standardized for the Korean population. Despite this, we included them in the pilot clinical trial as their validity has been supported by many studies and trials, as shown in Table 2.

We designated the control group in the clinical trial as a waitlist control group because it is considered unethical to withhold interventions from adolescents with ASD and SCD who require continuous treatment. Participants in both the intervention and control groups continued receiving their usual care from 4 weeks before the start of screening to study conclusion.

The sample size for this study was determined by reviewing previous research with similar participant diagnoses or intervention methodologies [33-36]. We constructed the protocol with a sample size of 40. However, as this was a pilot randomized clinical trial, we had no previous data for statistical calculation. The absence of statistical analytical justification for the sample size is a limitation of this study.

The frequency and duration of NDTx-01 use require further evidence to establish their optimal configuration. While intensive treatments have shown efficacy in various studies [66-68], the daily use of NDTx-01 may be perceived as burdensome, potentially affecting user compliance. We developed NDTx-01 as a 6-week intervention based on references from several studies and trials ranging from 4 to 12 weeks [34,35,69-73]. To sustain the effectiveness of NDTx-01, follow-up interventions are necessary. These concerns must be addressed in a confirmatory randomized clinical trial.

### Conclusions

Herein, we presented a clinical trial protocol to assess the efficacy and safety of a digital therapeutic, a new treatment designed to improve social cognition and communication in adolescents diagnosed with ASD and SCD. By analyzing the data, we intended to identify the primary outcome measure for assessing improvements in social cognition and communication skills, which would be crucial for a subsequent confirmatory clinical trial. Ultimately, this study laid the groundwork for the application of digital therapeutics in treating adolescents facing challenges in social communication and interaction.

## Abbreviations

ADI-R: Autism Diagnostic Interview–Revised
ASD: autism spectrum disorder
CGI-I: Clinical Global Impressions–Improvement
CGI-S: Clinical Global Impressions–Severity
FGI: focus group interview
FSIQ: full-scale intelligence quantity
K-SIPA: Korean version of the Stress Index for Parents of Adolescents
K-SSRS: Korean version of the Social Skills Rating System
K-VABS-II: Korean version of the Vineland Adaptive Behavior Scales, Second Edition
K-WISC-IV: Korean version of the Wechsler Intelligence Scale for Children–Fourth Edition
K-WISC-V: Korean version of the Wechsler Intelligence Scale for Children–Fifth Edition
PEERS: Program for the Education and Enrichment of Relational Skills
RCMAS-2: Revised Children's Manifest Anxiety Scale, Second Edition
SCD: social communication disorder
SCQ–Current Form: Social Communication Questionnaire–Current Form
SRS-2: Social Responsiveness Scale, Second Edition
VABS: Vineland Adaptive Behavior Scales
